# Occupational risks of COVID-19: a case-cohort study using health insurance claims data in Germany

**DOI:** 10.1186/s12889-024-20706-3

**Published:** 2024-11-21

**Authors:** Karla Romero Starke, René Mauer, Janice Hegewald, Ulrich Bolm-Audorff, Gabriela Brückner, Katrin Schüssel, Helmut Schröder, Andreas Seidler

**Affiliations:** 1 Institute and Policlinic of Occupational and Social Medicine, Faculty of Medicine, Technische Universität Dresden, Fetscherstraße 74, 01307 Dresden, Germany; 2 Institute for Medical Informatics and Biometry, Faculty of Medicine, Technische Universität Dresden, Dresden, Germany; 3 Division Work and Health, Federal Institute for Occupational Safety and Health (BAuA), Berlin, Germany; 4 AOK Research Institute (WIdO), Berlin, Germany

**Keywords:** SARS-CoV-2, COVID-19, Hospitalization risk, Work, Occupation

## Abstract

**Background:**

Studies on occupation and COVID-19 infection that cover a range of occupational groups and adjust for important confounders are lacking. This study aimed to estimate occupational risks of hospitalization with COVID-19 by taking into account sociodemographic factors and previous comorbidities.

**Methods:**

We applied a case-cohort design using workers insured with one of Germany’s largest statutory health insurers as a data source for occupational and demographical information as well as for information on comorbidities. Cox regression models with denominator weights for cases and controls assessed relative risks of hospitalization with COVID-19 in 2020.

**Results:**

The study consisted of 11,202 COVID-19 cases and 249,707 non-cases. After adjusting for age, sex, number of pre-existing comorbidities, and socioeconomic status, we found at least doubled risks for occupations in theology and church work (HR = 3.05; 95% CI 1.93–4.82), occupations in healthcare (HR = 2.74; 95% CI 2.46–3.05), for bus and tram divers (HR = 2.46; 95% CI 2.04–2.97), occupations in meat processing (HR = 2.16; 95% CI 1.57–2.98), and professional drivers in passenger transport (e.g. taxi drivers) (HR = 2.00; 95% CI 1.59–2.51). In addition, occupations in property marketing and management, social workers, laboratory workers, occupations in personal care (e.g. hairdressers), occupations in housekeeping and occupations in gastronomy all had statistically significantly increased risks compared to the reference population (administrative workers).

**Conclusions:**

We identified occupations with increased risks for hospitalization with COVID-19. For those having a doubled risk it can be assumed that COVID-19 diseases are predominantly occupationally related. By identifying high-risk occupations in non-healthcare professions, effective measures to prevent infections in the workplace can be developed, also in case of a future pandemic.

**Supplementary Information:**

The online version contains supplementary material available at 10.1186/s12889-024-20706-3.

## Background

Employees in certain occupational settings may be exposed to biological agents, and thus may have higher risks for infections than the general working population. This became apparent during the COVID-19 pandemic, when healthcare workers faced a disproportionate burden of the infection [1–3]. By September 2022, the German Statutory Accident Insurance and Prevention in the Health and Welfare Services, the compensation board of private health and social work providers, had already recognized more than 200,000 COVID-19 cases as occupational diseases [4]. In Germany, COVID-19 can be recognized as an occupational disease when the infection occurs at the workplace for healthcare, social sector, and laboratory workers, or for occupations with a comparably high infection risk (Occupational Disease Number 3101, OD3101) [5].

The exceptional circumstances of the COVID-19 pandemic, including the transmission of the virus during a long asymptomatic incubation period, may have also exposed occupational groups outside the healthcare sector to an increased risk of infection. Especially workers who could not work from home during the pandemic, may have also faced an increased risk of infection [6, 7]. These essential workers were employed, among others, in food production, cleaning, sales, public transport, logistics, law enforcement, and in geriatric and child care. Prior to the pandemic, it was shown that daycare workers have at least a doubled risk for certain infections compared to the general population [8–10]. A substantial increased risk of infection for certain occupations could lead to the recognition of COVID-19 as an occupational disease for those workers. Further, a better understanding of the risks of infection in non-healthcare professions will also help to develop effective measures to prevent infections in the workplace.

Several studies have investigated the occupational risk for a SARS-CoV-2 infection, but most have only included certain occupations in their analyses [6, 11–17] or the occupations have been grouped in broad categories [7, 18, 19], limiting the knowledge of risks associated with specific occupations. Further, many studies have not adjusted for important confounders, such as sex and age (for instance [12, 18, 20–24]). When using severe COVID-19 outcomes, such as hospitalization or mortality as a proxy for infection, it is also necessary to adjust for pre-existing comorbidities [25], and only a few studies have done this [6, 26]. To date, there has been no study in Germany analyzing specific occupational sub-groups, while adjusting for important confounders.

In the present study, we aimed to identify high-risk occupations among workers in Germany by examining hospitalizations with COVID-19 by occupational group. For this purpose, we used the data on workers insured in a large German statutory health insurance provider to build a case-cohort study for 2020, and calculated hazard ratios adjusted for sex, age, number of pre-existing comorbidities, and socioeconomic status (SES).

## Methods

### Study population

For this study, we used a case-cohort design, first presented by Prentice in 1986 [27]. In a case-cohort study, a random sample of an entire cohort is selected (the “sub-cohort”), and up to 100% of the cases are chosen. This necessitates only the collection of covariates for the sub-cohort and for the cases [27]. We chose the design because of increased flexibility, since the resulting sub-cohort can be used as a comparison for different outcomes, while having similar statistical power as nested case-control studies [28]. In our design, we included 100% of cases.

We analyzed anonymized insurance claims data from 2020 using the AOK (Allgemeine Ortskrankenkasse), a large statutory health insurance provider covering about one third of the German population. The base population included all persons aged 15 to 70 years and with employment subject to social insurance contributions in 2020. In order to identify pre-existing comorbidities, study participants had to be continuously enrolled with the health insurer in 2019 (the year before the start of the study). After considering the inclusion and exclusion criteria, there were 9,186,934 eligible insurees at the beginning of 2020. Initially, we had expected about 60,000 COVID-19 cases in 2020, and we targeted a 1:4 proportion of cases to non-cases. Therefore, we chose a sub-cohort of 250,000 insurees who were randomly selected from the eligible population (sampling fraction [α] = 2.72%). We defined cases as workers with a first hospital discharge diagnosis of a laboratory-confirmed COVID-19 infection (ICD U07.1!) documented in 2020. The code for the hospital discharge included person who may have died at the hospital.

### Exposure

The main exposure was the occupation at the start of 2020. Occupations were ascertained using the occupational coding scheme German Classification of Occupations (Klassifikation der Berufe or KldB 2010), developed by the German Federal Employment Agency and the German Federal Statistical Office [29]. The KldB 2010 has a hierarchical classification with five breakdown levels, starting with the more general occupational areas (1-digit codes), and increasing in detail and similarity among the included occupations (occupational main groups [2-digit codes], occupational groups [3-digit codes], occupational sub-groups [4-digit codes, used for this project], and occupational types [5-digit codes]). Our main analysis consisted of the selective evaluation of 24 distinctive occupational categories made up of several occupational sub-groups with comparable job tasks and comparable levels of work-related personal contacts. These occupational categories were classified by experienced occupational physicians (AS and UBA). We also evaluated each 4-digit KldB codes separately. For the reference group, we used administrative workers, as they were able to move to home office and would therefore be at a lower risk of acquiring COVID-19 through the occupation. Table S1 contains the list of occupational categories and their sub-groups.

### Covariates

We considered age, sex, number of comorbidities, and SES as confounders. We evaluated age in five-year categories, but used it as a metric variable by using the midpoints of each age category. It has been previously shown that by itself (without the effect of comorbidities which also increase with age and increases the risk of severe COVID-19 outcomes, the risk of severe outcomes increases linearly with age [30]. The prevalence of comorbidities varies between occupations, and comorbidities are associated with a severe course of COVID-19, according to a meta-analysis [25]. The results of the meta-analysis were validated in a study using the German population [31]. Based on the identified 19 conditions (Table S2), we took the number of these comorbidities for each participant in the analysis.

We estimated SES in two ways. The first was through the German Index of Socioeconomic Deprivation (GISD), an index generated at the district and municipality level based on educational, employment, and income data, developed by the Robert Koch Institute [32]. The index takes values between 0 (lowest deprivation) and 1 (highest deprivation). Since it is an aggregated marker, it may not reflect the individuals’ SES. Thus, we complemented it with the participants’ education, specifically, their vocational training in 2020. There were four categories: [1] no vocational training [2], vocational training [completion of recognized vocational training or master craftsman, technician or equivalent] [3], university degree program (diploma/master’s/state examination or doctorate), and [4] unknown status. Although our categorization has broader categories, it is compatible with the “Comparative Analyses of Social Mobility in Industrial Nations” (CASMIN) [33].

### Statistical analysis

To examine the association between occupation and hospitalization with COVID-19, we estimated hazard ratios (HRs) using weighted and stratified Cox regression models using death, end of insurance in the AOK or the change of, a break in or the end of the occupation, all in the year 2020, as censored observations. We defined the time to event by the number of months after the start of 2020 to the first outcome occurrence. The case-cohort design was performed according to the method described by Prentice [27]. As previously stated, a sub-cohort is randomly chosen from the base population, while taking all cases into the analysis. In this situation, cases are over-represented, and a weight correction must therefore be used. We applied Barlow’s approach of the denominator weights for cases and controls [34]. Cases outside the sub-cohort received a weight of 0 just before the event, and a weight of 1.0 starting at the time (month) of diagnosis. In the sub-cohort, we weighted non-cases as 1/α for all their follow-up time (in months), while cases received a weight of 1/α from the start of follow-up until just before their event time; at their event, cases received a weight of 1.0. As suggested by Barlow [35], we estimated confidence intervals using a robust variance estimator for the case-cohort design.

The main analysis consisted of three models with different confounder sets: model 1 adjusted for age and sex, model 2 additionally adjusted for the number of comorbidities, and model 3 included SES (GISD and individual education). We anticipated a large proportion of participants with an unknown educational status for the year 2020. Consequently, in a subsequent analysis, we used the last known educational status of these participants from 2011 to 2019. Furthermore, we expected a relatively large proportion of younger participants (< 30 years) to have not completed their vocational training. Recent data from the microcensus in Germany shows that 26% of people between 25 and 30 years have not completed a vocational training [36]. In the older age categories, the proportion is lower (11–18%). Categorizing younger participants who may still be in training into “no vocational training” could result in a SES classification that may not reflect their real situation. Therefore, in a further model, we replaced the SES category “no vocational training” with two categories: “<30 years and no vocational training”, and “≥30 years and no vocational training”.

In order to take spatial differences in the spread of COVID-19 into account, we included the federal state of residency as a random effect in the model. Although district-level data was available, this proved to be too fine for model stability, as there were often only a couple of participants per district. We considered occupational categories presented in Table S1 in the analyses as long as the sum of all cases in the occupational 4-digit sub-groups comprising the occupational group was at least 10. Otherwise, we included these persons in the category “other occupations” in the analyses. There were always three occupational groups considered in each analysis: [1] administrative workers (the reference group) [2], the considered occupational category/sub-group, and [3] other occupations.

All analyses were done in R version 4.3.2 [37].

## Results

### Sample characteristics

**Fig. 1:**
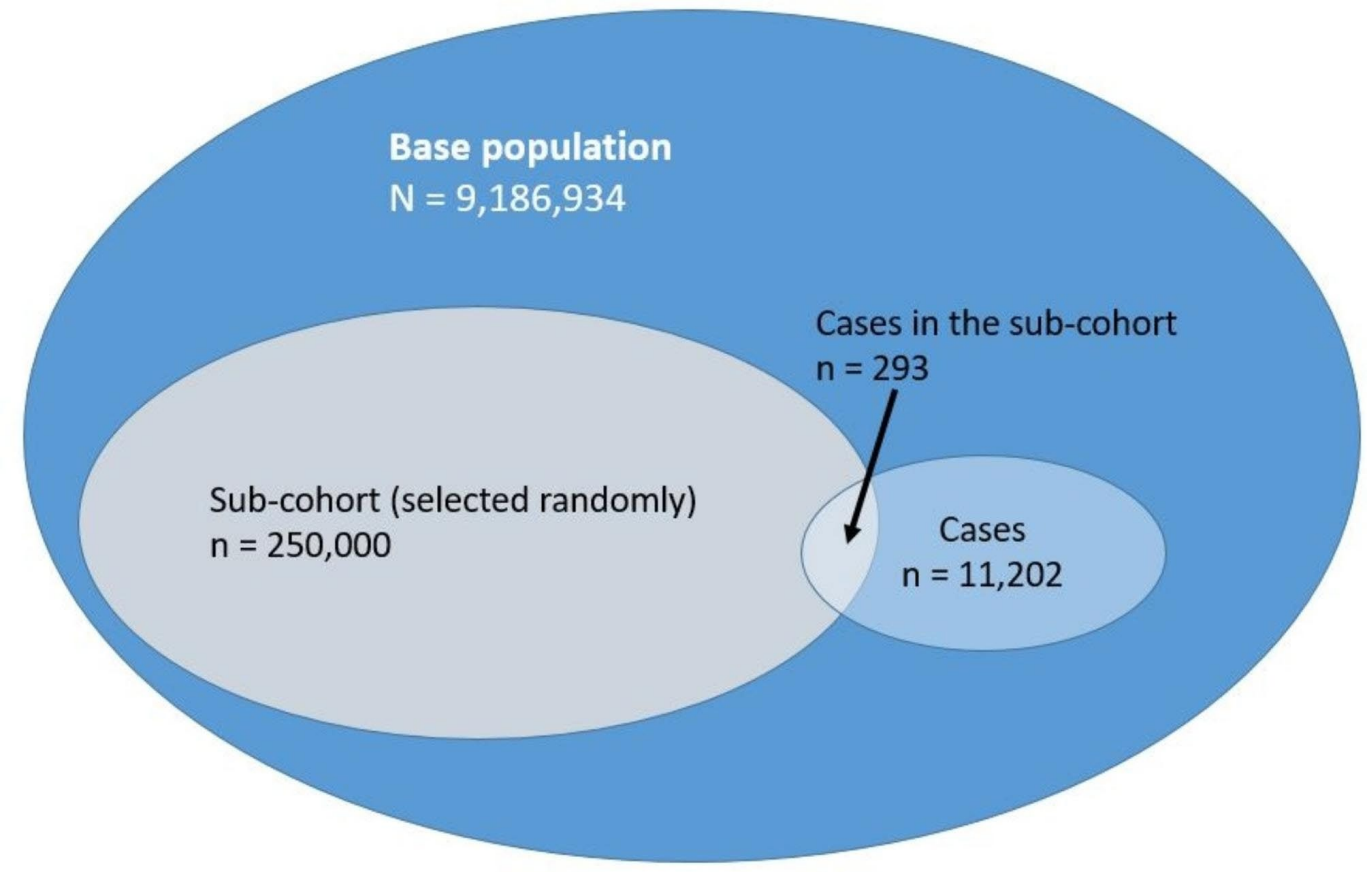
Graphical representation of the case-cohort study

There were 11,202 workers with a first hospital discharge diagnosis of a laboratory-confirmed COVID-19 infection, fulfilling our case definition. Their study characteristics, along with the non-cases (*n* = 249,707) in the sub-cohort are presented in Table 1. Figure 1 is a graphical representation of the numbers of participants in each group of the case cohort design. Cases were older than non-cases (48.3 years vs. 41.5 years) and had a higher percentage of men (59% vs. 53%). As well, cases were afflicted with more comorbidities on average than non-cases (0.42 vs. 0.17). There was a slightly lower percentage of workers with a vocational training or degree program for cases compared to the non-cases in the sub-cohort. Educational background in 2020 was unknown for 27.6% of cases and 23.0% of non-cases in the sub-cohort. The percentage of total workers with no vocational training in 2020 was 15.4%, and was highest for the youngest age groups (15–29 years). In the older age groups, the percentage of workers having no vocational training was relatively constant at 10–14% (Table S3).

**Table 1:** Characteristics of COVID-19 cases and sub-cohort non-cases

Variable	Cases *N* (%)	Non-cases in sub-cohort *N* (%)
Total	11,202 (100%)	249,707 (100%)
Age (years)		
Mean (SD)	48.31 (11.64)*	41.49 (13.00)*
15–19	96 (0.86%)	7216 (2.89%)
20–24	345 (3.08%)	20,491 (8.21%)
25–29	620 (5.53%)	27,932 (11.19%)
30–34	743 (6.63%)	31,565 (12.64%)
35–39	729 (6.51%)	27,773 (11.12%)
40–44	978 (8.73%)	24,758 (9.91%)
45–49	1437 (12.83%)	26,461 (10.60%)
50–54	2106 (18.80%)	32,271 (12.92%)
55–59	2341 (20.90%)	30,722 (12.30%)
60–64	1645 (14.68%)	18,591 (7.45%)
65–69	162 (1.45%)	1927 (0.77%)
Sex		
Male	6623 (59.12%)	132,978 (53.25%)
Female	4579 (40.88%)	116,729 (46.75%)
GISD Index		
Mean (SD)	0.50 (0.18)*	0.52 (0.17)*
Education (vocational training) in 2020		
No vocational training	1966 (17.55%)	38,546 (15.44%)
Vocational training	5751 (51.34%)	139,458 (55.85%)
Degree program	390 (3.48%)	14,369 (5.75%)
Unknown status	3095 (27.63%)	38,546 (22.96%)
Number of comorbidities		
Mean (SD)	0.42 (0.86)*	0.17 (0.51)*

*Refers to mean and standard deviation of continuous variable

### Occupational risk of hospitalization with COVID-19

**Fig. 2:**
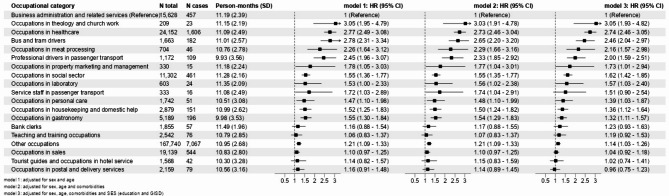
Hazard ratios (HRs) for hospitalization with COVID-19 for occupational categories described in Table S1, sorted by decreasing risks according to Model 3

Figure 2 presents the results for the regression modeling by occupational groups. Occupational groups with less than ten cases were included in the category “other occupations”; therefore, not all occupational groups in Table S1 are shown. Persons with occupations in theology and church work had the highest risk for hospitalization with COVID-19 (HR = 3.05; 95% CI 1.93–4.82), followed by occupations in healthcare (HR = 2.74; 95% CI 2.46–3.05), bus and tram drivers (HR = 2.46; 95% CI 2.04–2.97), occupations in meat processing (HR = 2.16; 95% CI 1.57–2.98), and professional drivers in passenger transport (HR = 2.00; 95% CI 1.59–2.51). Occupations in property marketing and management, occupations in the social welfare sector, laboratory workers, occupations in personal care, occupations in housekeeping and domestic help and in gastronomy all had statistically significantly increased risks of hospitalization ranging from a HR of 1.32 (gastronomy) to 1.73 (marketing and management). We also observed increased risks (HR 1.19 to 1.51) for service staff in passenger transport, bank clerks, and teaching/training occupations, but these categories did not reach statistical significance. The full model results are shown in Table S4.

Table S5 contains results for the occupations using 4-digit KldB codes. After adjustment for all confounders, occupations in theology had the highest risk of hospitalization (HR = 11.17; 95% CI 5.87–21.26). Several occupations in healthcare [e.g. non-specialized physicians, healthcare and nursing professions, professions in geriatric care], occupations in social services (professions in home and family care), bus and tram drivers, professions in technical aviation operations, service specialists in road and rail transport, professions in the production of building materials and in metal production, professions in the industrial foundry, meat processing, catering industry, and industrial ceramics had at least a doubled risk of hospitalization.

### Sensitivity analyses

Because having no vocational qualification was more common among the under-30s, we divided this educational category into “under-30s” and “over-30s”. The occupational relative risks in this analysis show no discernable differences to the main results (Table S6).

For workers whose educational information was missing for 2020, we replaced it with information available in the years 2011 to 2019. By doing so, we could account for 41% of the missing data, and the proportion of missing educational data decreased from 23.2 to 13.7%. The majority of workers (59%) with missing educational information for 2020 were still missing this information, 23.8% had vocational training, 24.9% had no vocational education, and 3.4% had a university degree using the data from 2011 to 2019. Table S7 shows the resulting relative risks using this updated educational data. The relative risks showed no noticeable difference to the main results.

We also depicted the distribution of cases by specific and unspecific case definitions (hospitalization with U07.1 and U07.2) for the occupations with the highest increased risks, as well as for the reference category (Figure S1). One can observe the first and second COVID-19 waves (Wave 1 from March to May; Wave 2 from end of September until after the end of 2020 [38]) clearly with the more accurate laboratory-confirmed U07.1 case definition. The “suspected case” U07.2 definition did not differentiate both waves, but rather the cases remained relatively constant after the first wave.

## Discussion

This study investigated the risk of hospitalization with COVID-19 in workers using health insurance claims data. After adjusting for age, sex, number of comorbidities, and SES, we found that relative to administrative workers, occupations in theology and church work, occupations in healthcare, occupations in meat processing, professional drivers in passenger transport, and bus and tram drivers had at least a doubled risk of hospitalization. Other occupations with an increased risk of hospitalization include occupations in the social welfare sector, occupations in property marketing and management, laboratory workers, occupations in personal care, in housekeeping and domestic health, and in gastronomy. In addition, occupations in property management, service staff in passenger transport, bank clerks, and teachers had increased risks, but they were not statistically significant.

After adjustment for number of comorbidities, the relative risks did not change considerably. Adjusting for educational status and GISD tended to result in lower risks, but there were exceptions. These relative risks were robust to the sensitivity analyses on educational status.

### Comparison to other studies

When investigating healthcare workers, studies have consistently found that they had among the highest risks compared to the reference population [6, 7, 19, 26, 39–43], although Magnusson et al. 2021 [41] observed an increase in the first, but not in the second wave. Similar to our study, studies including social care workers observed some of the highest risks for this occupational group [26, 40, 42]. An exception was Billingsley and colleagues [11], who found lower risks for social care workers compared to the reference population (Information technology [IT]/administration) only after adjusting for SES. We also found statistically significant increased risks for laboratory workers. However, studies investigating risks for this particular occupational group were scarce, perhaps because laboratory workers may have been grouped together with healthcare workers. One study found a more than doubled risk of infection for laboratory workers compared to pathologists [44], but this study was based on a voluntary reporting system, and the presence of selection bias cannot be excluded. Due to contact with positive samples, it is conceivable that laboratory workers are at an increased risk for infection.

Like the present study, Billingsley and authors [11] also found higher mortality risks for taxi and bus drivers compared to skilled workers in economics, IT and administration, but the relative risks were not statistically significant, perhaps due to lack of study power. Magnusson et al. 2021 [41] found statistically significant increased risks for COVID-19 infection for occupations in personal transport (including taxi drivers) and for bus and tram drivers. Nafilyan and colleages similarly found increased risks for taxi drivers and chauffeurs, bus and coach drivers, and van drivers, compared to corporate managers and directors [26]. Finally, the UK Industrial Injuries Advisory Council (IIAC), also described more than two-fold risks for taxi drivers and chauffeurs, as well as for bus drivers, compared to the general working population [42].

Workers in the meat processing industry had among the highest hazard ratios. Starting in the Spring of 2020, in Germany there were several well-documented COVID-19 outbreaks in meat processing plants [45, 46]. Other countries also had similar experiences [47–49]. Like our study, a study from the USA observed a more than doubled increased risk of COVID-19 positivity for workers in the meatpacking industry compared to all others [14]. Factors like cold temperatures, crowded conditions, and a loud environment (conducing workers to speak loudly over the noise) contribute to an environment conducive to higher transmission/infection rates [50].

Furthermore, we found almost 40% increased risks for workers in personal care, mainly influenced by increased risks for hairdressers (Table S4). Other studies likewise found increased risks for “caring personal services” (33% increased risks) compared to managers and directors [26] and at least doubled risks for hairdressers and barbers, compared to the general working population [42]. With regards to housekeeping/domestic help we found 36% increased risks for infection, similar to the IIAC position paper [42], which observed at least doubled risks for cleaners. However, Billingsley and colleagues [11] observed lowered risks for cleaners when adjusting for socioeconomic status, while Nafilyan et al. only observed statistically non-significant relative risks when further adjusting by household size and ethnicity [26]. The heterogeneity of results for this group may be explained by the heterogeneity of the occupational sub-group itself, which includes individuals caring for the elderly or sick (Table S1). Like our paper, several studies comparing occupations to the working population found increased risks for occupations in gastronomy (waiters/waitresses, caterers, bartenders) [41, 42], but Nafilyan found either statistically non-significant relative risks or lowered risks after adjusting for socioeconomic characteristics and ethnicity [26].

To our knowledge, this is the first study to report high relative risks for infection in occupations in theology and churchwork. The increased risk is driven by occupations in theology (e.g. Protestant pastor, Catholic priest), which have a 11 times higher risk compared to administrative workers. Pastors and priests were an important pillar for moral support during COVID-19. Although churches faced closures during 2020, including wedding/baptism cancellations and restrictions on funeral services, there were phases when churches were open [51]. Online spiritual guidance was offered, but sometimes personal contact took place. Moreover, clergy continued to accompany the sick and dying during the pandemic [52].

### Strengths and limitations

The main strengths of this study are its prospective nature and its efficient design. It used data from one of the largest statutory health insurance providers in Germany, covering about one-third of insurees. The use of secondary health claims data reduced the possibility of selection bias, since all AOK insurees in employment were included in the analysis. The outcome was assessed objectively, since it was based on hospital records. We chose to evaluate hospitalization, rather than sick leave, as it would be less affected by the lockdown situation. The year 2020 was characterized by lockdowns, school closures and business closings. If, as an example, a waiter was at home because its employer (restaurant) was forced to close due to the lockdown, and he/she developed COVID-19, it is likely he/she may not have filed for sick leave since he/she was at home anyway. Furthermore, it was possible to obtain a sick leave from a doctor via telephone if a respiratory illness was present. If a doctor suspected a COVID-19 infection, this could have been coded as ICD U07.2! in the sick leave, leaving uncertainty about the true cause of infection. Therefore, we judged an analysis using hospitalization less prone to bias compared to using sick leaves.

Another major strength in our study is the adjustment for confounders age, sex, number of comorbidities, and SES. Although there have been several studies investigating occupational risks for COVID-19 infections, the majority have not adjusted for all of these confounders [7, 11, 13, 14, 16–24, 39–43, 53], with the exception of Nafilyan et al. 2021 [26] and Nwaru et al. 2022 [6].

We adjusted for SES using aggregated data, and to minimize residual confounding, we further adjusted for SES with individual information education. Even though more than 20% of the latest education data from the year 2020 was missing, we could replace a large proportion of the missing education data through information obtained in the years 2011 to 2019, reducing missing data to 13.7%. Using this updated data, the relative risks were similar to those in the main analysis, which illustrates the robustness of the results.

Adjustment for SES in the context of our research question merits discussion. On the one hand, there may be differences in SES within and between occupational groups that should be considered. On the other hand, occupation is an important aspect of SES, and adjustment for SES may lead to an “overadjustment”; the relative risks may therefore be underestimated. Billingsley et al. consistently found decreased risks when adjusting for SES [11]. In most cases, we observed a tendency for reduced risks when adjusting for SES, although in most of the cases the relative risks remained statistically significant.

One study found differing risks (not consistently increased or decreased) when considering different cultural or ethnical groups [41]. We had no information on cultural groups in our dataset, so confounding in this respect cannot be excluded. In addition, undetected confounding due to other unknown non-occupational factors might be present.

This study did not consider possible mediators, such as the use of public transport, in the analysis. Our aim, similar to most epidemiological studies of this nature, was to study the total effect of the exposure on the outcome. In principle, the use of public transport could have differed between different occupational groups. However, we do not expect employees in occupations with an increased COVID-19 risk (e.g. bus drivers, taxi drivers, priests) to use public transport considerably more often than employees in other occupations. We therefore do not t expect mediation by the use of public transport to undermine the direct effect of the occupation on COVID-19 infection.

In addition, the comorbidities we considered differ in their effect on severe outcomes [25]. However, we simplified this in the models, essentially assigning the same weights for all chronic conditions by considering the number of comorbidities, and not the specific conditions. Nonetheless, our study has taken into account the most important comorbidities impacting severe outcomes [25].

We used hospitalization as the outcome, which is less frequent than a COVID-19 infection. Some of the occupations may not have had enough power to reach statistical significance. Nonetheless, hospitalization is an objective and reliable outcome less influenced by testing capacities during the pandemic. The use of the laboratory-confirmed U07.1 definition is specific and robust, even though an underestimation of the actual cases may have occurred due to the lack of testing at the beginning of the pandemic. However, the additional use of the suspected-case U07.2 would have been too inexact, as the waves were not visible in the time trend analysis.

Finally, our results were based on data from a large statutory health insurance. Some workers may have been underrepresented, such as self-employed persons and some civil officials (who are exempt from compulsory statutory health insurance and rather have private insurance). Civil officials in Germany who are excluded from compulsory statutory health insurance include police, firefighters, teachers, judges, and lawyers. Moreover, priests and other theologians in Germany are often recognized public servants with special benefits (“Kirchenbeamte”), and they may be accordingly privately insured. High-earners in different occupations (e.g. doctors, dentists, lawyers) may also be privately insured. A recent analysis of the AOK insurees showed that it has an above-average proportion of insured persons from the agricultural sector, the traffic and transportation sector and the construction industry [54]. However, the service branch, the energy, water, waste, and mine branch, the education branch, the health and social service sector, the metal industry, commerce, public administration, and manufacturing industry are well-represented (their membership in the AOK ranging from 30 to 46% of their total sector) [55]. Lower proportions of AOK-insured persons in certain occupational groups do not necessarily result in bias, unless those insured by the AOK are systematically different in terms of professional contacts than those not insured by the AOK.

### Implications for prevention and public policy

Prevention strategies could be introduced or reinforced in the identified high-risk occupations, in case of a future pandemic with a similar mode of transmission. These include known measures such as social distancing, mask wearing, airing rooms, and frequent handwashing.

The doubling risk as a rule of thumb is important in many countries for the introduction or identification of occupational diseases, even if the doubling risk can only be equated with a causation probability of 50% under specific boundary conditions [56–58]. Nonetheless, following this rule of thumb, we could identify occupational groups with at least a doubled risk of hospitalization with COVID-19. These include occupations in theology, service specialists in road and rail transport, workers in the production of building materials and metal production, and bus and tram drivers. In addition, certain healthcare and social service workers also had more than a doubled risk of hospitalization. The Occupational Diseases Ordinance stipulates that besides workers in healthcare, social sector and in laboratories, other occupations may be eligible for OD3101 if the risk of infection was similar to the three aforementioned occupations. To date, other occupational groups have not been eligible for an occupational disease recognition in Germany, but have been recognized as work accidents (e.g. meat packers). Although the benefits for work accidents and occupational diseases are the same or very similar for infectious diseases, it is not easy to prove a work accident for COVID-19, as intensive occupational contact with the infectious person(s) (“index case”) should be demonstrated [59], which is not always easy to do. This study underlines the possibility for eligibility of the previously named occupations as occupational diseases using risk estimates for the year 2020. Starting the implementation of vaccines starting in December 2020 and in 2021, the occupational risk estimates could have changed (for instance, in healthcare workers, for whom vaccination was compulsory).

## Conclusions

After adjustment for age, sex, number of comorbidities, and SES we identified several occupational groups with a substantially higher risk for COVID-19 hospitalization. These workers could be eligible to recognition as occupational diseases if infected with COVID-19.

## Electronic supplementary material

Below is the link to the electronic supplementary material.

Supplementary Material 1

## Data Availability

The data that support the findings of this study are available from WiDO but restrictions apply to the availability of these data, which were used under license for the current study, and so are not publicly available. Data availability is contingent on the permission of WiDO.

## Abbreviations

AOK: Allgemeine Ortskrankenkasse
CASMIN: Comparative Analyses of Social Mobility in Industrial Nations
COVID-19: Coronavirus disease 2019
GISD: German Index of Socioeconomic Deprivation
HR: Hazards Ratio
ICD: International Classification of Diseases
IIAC: Industrial Injuries Advisory Council
IT: Information technology
KldB 2010: German Classification of Occupations (Klassifikation der Berufe 2010)
OD: Occupational disease
SARS-CoV-2: Severe Acute Respiratory Syndrome Coronavirus-2
SES: Socioeconomic status
UK: United Kingdom
