# Supplementary material for: Occupational risks of COVID-19: a case-cohort study using health insurance claims data in Germany
Source: BMC Public Health. 2024 Nov 21;24:3235. doi: 10.1186/s12889-024-20706-3 (PMC11580653; doi:10.1186/s12889-024-20706-3)

| **KldB2010 classification** | **Occupational sub-groups** | **KldB2010 classification** | **Occupational sub-groups** |
| --- | --- | --- | --- |
| **Category 1 - Occupations in healthcare** | | | |
| 5332 | Professions in health supervision and hygiene monitoring | 8138 | Occupations in health care and nursing (other specific job description) |
| 5334 | Occupations in disinfection and pest control | 8140 | Doctors (without specialization) |
| 8110 | Medical assistants (without specialisation) | 8141 | Specialists in pediatrics and adolescent medicine |
| 8111 | Dental assistants | 8142 | Specialists in internal medicine |
| 8113 | Orthoptists | 8143 | Specialists in surgery |
| 8118 | Medical assistant (other specific job description) | 8144 | Specialists in the fields of skin diseases, sensory and sexual organs |
| 8122 | Medical-technical professions in functional diagnostics | 8145 | Specialists in anesthesiology |
| 8123 | Medical-technical professions in radiology | 8146 | Specialists in neurology, psychiatry, psychotherapy and psychosomatic medicine |
| 8130 | Healthcare and nursing professions (without specialization) | 8147 | Dentists and orthodontists |
| 8131 | Professions in specialized nursing | 8148 | Doctors (other specific job description) |
| 8132 | Professions in specialized pediatric nursing | 8149 | Managers - human medicine and dentistry |
| 8133 | Professions in surgical/medical technical assistance | 8163 | Professions in non-medical psychotherapy |
| 8134 | Professions in the rescue service | 8171 | Professions in physiotherapy |
| 8135 | Professions in obstetrics and maternity care | 8172 | Occupations in occupational therapy |
| 8173 | Professions in speech therapy | 8180 | Pharmacists |
| 8174 | Professions in music and art therapy | 8182 | Professions in pharmaceutical technical assistance |
| 8175 | Professions in medicine and homeopathy | 8188 | Occupations in pharmacy (other specific job description) |
| 8176 | Professions in diet and nutrition therapy | 8210 | Professions in geriatric care (without specialization) |
| 8177 | Podiatrists | 8218 | Occupations in geriatric care (other specific occupation) |
| 8178 | Professions in non-medical therapy and medicine (other specific activity) |  |  |
| 8179 | Managers - Non-medical therapy and medicine | 8223 | Professions in nutritional counselling |
| **Category 2 - Occupations in the social welfare sector** | | | |
| 8311 | Professions in childcare and education | 8314 | Professions in home and family care |
| 8312 | Professions in social work and social pedagogy | 8315 | Professions in social, educational and addiction counselling |
| 8313 | Professions in curative education and special needs education |  |  |
| **Category 3 - Occupations in laboratory** | | | |
| 8121 | Medical-technical professions in the laboratory | 8254 | Professions in dental technology |
| **Category 4 - Service staff in passenger transport** | | | |

Supplementary Table S1. Occupational categories and corresponding occupational sub-groups considered in main analysis

| 5140 | Service staff in passenger transport (without specialization), e.g. ticket inspector, ticket seller, flight attendant | 5142 | Service specialists in air transport, e.g. purser/purserette, ground steward/stewardess |
| --- | --- | --- | --- |
| 5141 | Service specialists in road and rail transport, e.g. travel advisor (rail), train attendant | 5143 | Service specialists in shipping (ship's steward/stewardess) |
| **Category 5 - Professional drivers in passenger transport** | | | |
| 5211 | Professional drivers (passenger transport/car) (company car driver, taxi driver) |  |  |
| **Category 6 - Bus and tram drivers** | | | |
| 5213 | Bus and tram drivers |  |  |
|  |  |  |  |
| **Category 7 - Firefighters, occupations in law enforcement, court and the prison system** | | | |
| 5313 | Professions in fire protection, e.g. professional firefighter | 5323 | Professions in the judicial system |
| 5321 | Professions in the police service | 5324 | Professions in the prison service |
| 5322 | Professions in the criminal investigation service |  |  |
| **Category 8 - Occupations in property marketing and management** | | | |
| 6131 | Professions in property marketing and management |  |  |
| **Category 9 - Occupations in sales** | | | |
| 62xx | Professions in sales |  |  |
| **Category 10 - Tourist guides and occupations in hotel service** | | | |
| 6314 | Tour and tourist guides | 6322 | Professions in hotel service, e.g. receptionist (hotel) |
| **Category 11 - Occupations in gastronomy** | | | |
| 6330 | Professions in catering service (without specialization), e.g. waiter/waitress | 6332 | Bartenders |
| 6331 | Professions in system catering, e.g. catering agent |  |  |
| **Category 12 - Bank clerks** | | | |
| 7211 | Bank clerks, e.g. customer advisor (bank, savings bank), loan officer |  |  |
| **Category 13 - Occupations in personal care** | | | |
| 8231 | Professions in the hairdressing trade | 8233 | Tattooists and piercers |
| 8232 | Professions in cosmetics | 8234 | Professions in make-up artistry |
| **Category 14 - Occupations in medical, orthopedic, and rehabilitation technology** | | | |
| 8251 | Professions in orthopedic and rehabilitation technology, e.g. orthopedic mechanic | 8253 | Professions in hearing aid acoustics |
| 8252 | Professions in optometry |  |  |
| **Category 15 - Occupations in housekeeping and domestic help** | | | |
| 8321 | Housekeeping professions, e.g. domestic helpers, some of whom also care for the elderly and sick |  |  |
| **Category 16 - Occupations in theology and church work** | | | |
| 8331 | Professions in theology, e.g. Protestant pastor, Catholic priest | 8338 | Professions in theology and parish work (other specific job description), e.g. church sexton, sacristan |
| 8332 | Professions in parish work, e.g. parish assistant/parish deacon |  |  |
| **Category 17 - Teaching and training occupations** | | | |
| 84XX | Teaching and training occupations |  |  |
| **Category 18 - Occupations in journalism** | | | |
| 9241 | Editors and journalists |  |  |
| **Category 19 - Occupations in music** | | | |
| 941X | Music, singing and conducting activities, e.g. philharmonic orchestra |  |  |
| **Category 20 - Occupations in acting and dance** | | | |
| 942X | Professions in acting, dance and the art of movement |  |  |
| **Category 21 - Occupations in meat processing** | | | |
| 2923 | Occupations in meat processing |  |  |
| **Category 22 - Occupations in postal and delivery services** | | | |
| 5132 | Occupations in postal and delivery services |  |  |
| **Category 23- Other occupations** | | | |
| All other occupations not mentioned in this table (occupational categories on this table which did not have at least 10 cases were added to this group) | | | |
| **Category 24 – Business administration and related services (reference group)** | | | |
| 732X | Administration (excluding 7321 “Professions in social administration and insurance” and 7322 “Administrative professions in the social and healthcare sector”) | 714X | Occupations in office and secretarial services |

Supplementary Table S2. Comorbidities included in the analysis

| Hemato-oncological diseases with therapy | Atrial fibrillation and atrial flutter |
| --- | --- |
| Metastasized solid tumor diseases | Interstitial lung disease |
| Dementia | Coronary heart disease |
| Heart failure | Severe mental illness |
| Dialysis | Diabetes mellitus Type I and II |
| Solid cancer with therapy | COPD and other severe lung diseases |
| Cirrhotic and severe liver diseases | Cerebrovascular diseases |
| Down’s syndrome | Obesity |
| Chronic renal insufficiency | Neurological Diseases |
| Organ transplantation |  |

Supplementary Table S3. Description of education (vocational training) by age group for the case-cohort study

| **Age Group (years)** | **Vocational training** | **N** | **%** |
| --- | --- | --- | --- |
| 15-19 | No vocational training | 5664 | 77,5 |
|  | Vocational training | 540 | 7,4 |
|  | University degree program | 7 | 0,1 |
|  | Unknown status | 1101 | 15,1 |
| 20-24 | No vocational training | 7333 | 35,2 |
|  | Vocational training | 9233 | 44,3 |
|  | University degree program | 417 | 2 |
|  | Unknown status | 3853 | 18,5 |
| 25-29 | No vocational training | 3719 | 13 |
|  | Vocational training | 16029 | 56,1 |
|  | University degree program | 2829 | 9,9 |
|  | Unknown status | 5975 | 20,9 |
| 30-34 | No vocational training | 3149 | 9,7 |
|  | Vocational training | 18462 | 57,1 |
|  | University degree program | 3526 | 10,9 |
|  | Unknown status | 7171 | 22,2 |
| 35-39 | No vocational training | 2967 | 10,4 |
|  | Vocational training | 15882 | 55,7 |
|  | University degree program | 2550 | 8,9 |
|  | Unknown status | 7103 | 24,9 |
| 40-44 | No vocational training | 2917 | 11,3 |
|  | Vocational training | 14226 | 55,3 |
|  | University degree program | 1602 | 6,2 |
|  | Unknown status | 6991 | 27,2 |
| 45-49 | No vocational training | 3318 | 11,9 |
|  | Vocational training | 16041 | 57,5 |
|  | University degree program | 1103 | 4 |
|  | Unknown status | 7436 | 26,7 |
| 50-54 | No vocational training | 4095 | 11,9 |
|  | Vocational training | 20982 | 61 |
|  | University degree program | 984 | 2,9 |
|  | Unknown status | 8316 | 24,2 |
| 55-59 | No vocational training | 4314 | 13 |
|  | Vocational training | 20431 | 61,8 |
|  | University degree program | 921 | 2,8 |
|  | Unknown status | 7397 | 22,4 |
| 60-64 | No vocational training | 2753 | 13,6 |
|  | Vocational training | 12409 | 61,3 |
|  | University degree program | 697 | 3,4 |
|  | Unknown status | 4377 | 21,6 |
| 65-69 | No vocational training | 283 | 13,5 |
|  | Vocational training | 974 | 46,6 |
|  | University degree program | 123 | 5,9 |
|  | Unknown status | 709 | 33,9 |

Suplementary Table S4. Risk of hospitalization with COVID-19 by occupational category

| **Variable** | **Category** | **KldB 2010 (applicable only for occcupational categories)** | **HR* (95% CI)** | **HR** (95% CI)** | **HR† (95% CI)** |
| --- | --- | --- | --- | --- | --- |
| Occupational category | Business administration and related services | 714x, 732x, !7321, !7322 | 1 (Reference) | 1 (Reference) | 1 (Reference) |
|  | Occupations in theology and church work | 8331, 8332, 8338 | 3.05 (1.95 - 4.79) | 3.03 (1.91 - 4.78) | 3.05 (1.93 - 4.82) |
|  | Occupations in healthcare | 5332, 5334, 8110, 8111, 8113, 8118, 8122, 8123, 8130, 8131,  8132, 8133, 8134, 8135, 8138, 8140, 8141, 8142, 8143, 8144, 8145, 8146, 8147, 8148, 8149, 8163, 8171, 8172, 8173, 8174,  8175, 8176, 8177, 8178, 8179,  8180, 8182, 8188, 8210, 8218, 8223 | 2.77 (2.49 - 3.08) | 2.73 (2.46 - 3.04) | 2.74 (2.46 - 3.05) |
|  | Bus and tram drivers | 5213 | 2.78 (2.31 - 3.34) | 2.65 (2.20 - 3.20) | 2.46 (2.04 - 2.97) |
|  | Occupations in meat processing | 2923 | 2.26 (1.64 - 3.12) | 2.29 (1.66 - 3.16) | 2.16 (1.57 - 2.98) |
|  | Professional drivers in passenger transport | 5211 | 2.45 (1.96 - 3.07) | 2.33 (1.85 - 2.92) | 2.00 (1.59 - 2.51) |
|  | Occupations in property marketing and management | 6131 | 1.78 (1.05 - 3.03) | 1.77 (1.04 - 3.01) | 1.73 (1.01 - 2.94) |
|  | Occupations in the social welfare sector | 8311, 831 , 8313, 831 , 8315 | 1.55 (1.36 - 1.77) | 1.55 (1.35 - 1.77) | 1.62 (1.42 - 1.85) |
|  | Occupations in the laboratory | 8121, 8254 | 1.53 (1.00 - 2.33) | 1.56 (1.02 - 2.38) | 1.57 (1.03 - 2.40) |
|  | Service staff in passenger transport | 5140, 5141, 5142, 5143 | 1.72 (1.03 - 2.89) | 1.74 (1.04 - 2.91) | 1.51 (0.90 - 2.54) |
|  | Occupations in personal care | 8231, 8232, 8233, 8234 | 1.47 (1.10 - 1.98) | 1.48 (1.10 - 1.99) | 1.39 (1.03 - 1.87) |
|  | Occupations in housekeeping and domestic help | 8321 | 1.52 (1.25 - 1.83) | 1.50 (1.24 - 1.82) | 1.36 (1.12 - 1.64) |
|  | Occupations in gastronomy | 6330, 6331, 6332 | 1.55 (1.30 - 1.84) | 1.54 (1.29 - 1.83) | 1.32 (1.11 - 1.57) |
|  | Bank clerks | 7211 | 1.16 (0.88 - 1.54) | 1.17 (0.88 - 1.55) | 1.23 (0.93 - 1.63) |
|  | Teaching and training occupations | 84xx | 1.06 (0.83 - 1.37) | 1.07 (0.83 - 1.37) | 1.19 (0.92 - 1.53) |
|  | Other occupations |  | 1.21 (1.09 - 1.33) | 1.21 (1.09 - 1.33) | 1.14 (1.03 - 1.26) |
|  | Occupations in sales | 62xx, !6219 | 1.10 (0.97 - 1.25) | 1.10 (0.97 - 1.25) | 1.04 (0.92 - 1.18) |
|  | Tourist guides and occupations in hotel service | 6314 6322 | 1.14 (0.82 - 1.57) | 1.15 (0.83 - 1.59) | 1.02 (0.74 - 1.41) |
|  | Occupations in postal and delivery services | 5132 | 1.16 (0.91 - 1.48) | 1.14 (0.89 - 1.45) | 0.96 (0.75 - 1.23) |
| Sex | Men | - | 1 (Reference) | 1 (Reference) | 1 (Reference) |
|  | Women | - | 0.78 (0.75 - 0.81) | 0.81 (0.78 - 0.84) | 0.82 (0.79 - 0.85) |
| Age (per 5 years) |  | - | 1.04 (1.04 - 1.05) | 1.04 (1.04 - 1.04) | 1.04 (1.04 - 1.04) |
| Comorbidies (per number of comorbidities) |  | - | - | 1.43 (1.40 - 1.46) | 1.42 (1.39 - 1.46) |
| GISD index |  | - | - | - | 0.61 (0.52 - 0.72) |
| Educational level | No vocational training | - | - | - | 1 (Reference) |
|  | Vocational training | - | - | - | 0.68 (0.64 - 0.72) |
|  | University degree program | - | - | - | 0.57 (0.51 - 0.63) |
|  | Unknown status | - | - | - | 0.94 (0.88 - 1.00) |

*Model 1: adjusted for age and sex

**Model 2: adjusted for age, sex, and number of comorbidities

†Model 3: adjusted for age, sex, number comorbidities, and SES (German Index of Socioeconomic Deprivation and educational status )

Supplementary Table S5. Risk of hospitalization due to COVID-19 by occupation

| **KldB Code** | **Occupation** | **N (total)** | **N (cases)** | **Person-months (SD)** | **HR^1^ (95% CI)** | **HR^2^ (95% CI)** | **HR^3^ (95% CI)** |
| --- | --- | --- | --- | --- | --- | --- | --- |
| 714x, 732x, !7321, !7322 | Business administration and related services | 15628 | 457 | 11.19 (2.39) | 1 (Reference) | 1 (Reference) | 1 (Reference) |
| 8331 | Occupations in theology | 62 | 17 | 10.35 (2.75) | 10.95 (6.05 - 19.81) | 9.95 (5.17 - 19.16) | 11.17 (5.87 - 21.26) |
| 8140 | Physicians (without specialization) | 247 | 22 | 10.70 (2.77) | 4.32 (2.72 - 6.86) | 4.30 (2.69 - 6.88) | 5.50 (3.42 - 8.86) |
| 8130 | Healthcare and nursing professions (without specialization) | 7719 | 630 | 11.13 (2.42) | 3.21 (2.84 - 3.64) | 3.18 (2.81 - 3.61) | 3.20 (2.83 - 3.63) |
| 8210 | Professions in geriatric care (without specialization) | 7250 | 596 | 10.97 (2.63) | 3.18 (2.80 - 3.61) | 3.10 (2.73 - 3.52) | 3.01 (2.65 - 3.42) |
| 8131 | Professions in specialised nursing | 492 | 38 | 11.29 (2.22) | 2.73 (1.92 - 3.87) | 2.72 (1.91 - 3.85) | 2.85 (2.01 - 4.04) |
| 5141 | Service specialists in road and rail transport | 113 | 10 | 11.18 (2.27) | 3.00 (1.54 - 5.84) | 2.92 (1.49 - 5.70) | 2.65 (1.35 - 5.20) |
| 8314 | Professions in home and family care | 750 | 60 | 10.85 (2.74) | 2.76 (2.08 - 3.67) | 2.72 (2.04 - 3.61) | 2.63 (1.97 - 3.49) |
| 2122 | Professions in the production of building materials | 285 | 27 | 11.22 (2.13) | 2.59 (1.71 - 3.92) | 2.58 (1.70 - 3.91) | 2.52 (1.66 - 3.83) |
| 5213 | Bus and tram drivers | 1663 | 182 | 11.01 (2.57) | 2.78 (2.31 - 3.34) | 2.65 (2.20 - 3.20) | 2.46 (2.04 - 2.97) |
| 2410 | Occupations in metal production (without specialization) | 152 | 14 | 10.99 (2.62) | 2.69 (1.53 - 4.74) | 2.69 (1.52 - 4.75) | 2.44 (1.38 - 4.30) |
| 8139 | Supervisors and managers - healthcare and nursing, emergency services and obstetrics | 291 | 20 | 11.01 (2.59) | 2.30 (1.44 - 3.67) | 2.22 (1.39 - 3.56) | 2.35 (1.47 - 3.77) |
| 5112 | Professions in technical aviation operations | 115 | 11 | 11.17 (2.23) | 2.59 (1.37 - 4.93) | 2.60 (1.37 - 4.94) | 2.33 (1.22 - 4.42) |
| 8134 | Professions in the rescue service | 477 | 26 | 11.36 (2.16) | 2.24 (1.48 - 3.38) | 2.24 (1.48 - 3.39) | 2.27 (1.50 - 3.44) |
| 2413 | Professions in the industrial foundry | 370 | 32 | 11.21 (2.29) | 2.33 (1.59 - 3.41) | 2.40 (1.64 - 3.52) | 2.21 (1.50 - 3.24) |
| 2923 | Professions in meat processing | 704 | 46 | 10.76 (2.78) | 2.26 (1.64 - 3.12) | 2.29 (1.66 - 3.16) | 2.16 (1.57 - 2.98) |
| 6331 | Professions in the catering industry | 346 | 21 | 10.52 (3.07) | 2.67 (1.70 - 4.20) | 2.58 (1.63 - 4.08) | 2.14 (1.35 - 3.39) |
| 2141 | Professions in industrial ceramics (process and plant engineering) | 187 | 15 | 10.90 (2.73) | 2.41 (1.40 - 4.15) | 2.25 (1.29 - 3.92) | 2.10 (1.20 - 3.65) |
| 8110 | Medical assistants (without specialization) | 3180 | 139 | 11.18 (2.36) | 2.05 (1.69 - 2.50) | 2.04 (1.68 - 2.48) | 2.06 (1.70 - 2.51) |
| 5211 | Professional drivers (passenger transport/car) | 1172 | 109 | 9.93 (3.56) | 2.45 (1.96 - 3.07) | 2.33 (1.85 - 2.92) | 2.00 (1.59 - 2.51) |
| 8172 | Occupations in occupational therapy | 348 | 16 | 11.28 (2.17) | 1.91 (1.14 - 3.20) | 1.86 (1.10 - 3.12) | 1.94 (1.15 - 3.26) |
| 2920 | Professions in food production (without specialization) | 1859 | 117 | 10.73 (2.90) | 2.16 (1.75 - 2.68) | 2.16 (1.74 - 2.67) | 1.91 (1.54 - 2.37) |
| 8412 | Secondary school teachers | 414 | 20 | 10.68 (2.99) | 1.54 (0.97 - 2.45) | 1.60 (1.00 - 2.54) | 1.90 (1.19 - 3.04) |
| 2411 | Professions in metallurgy | 149 | 11 | 11.23 (2.06) | 2.01 (1.07 - 3.77) | 1.87 (0.99 - 3.52) | 1.87 (0.99 - 3.52) |
| 3211 | Professions in concrete and reinforced concrete construction | 420 | 24 | 10.72 (2.94) | 1.88 (1.22 - 2.89) | 2.00 (1.30 - 3.07) | 1.86 (1.21 - 2.85) |
| 8121 | Medical-technical professions in the laboratory | 338 | 15 | 11.32 (2.13) | 1.72 (1.01 - 2.92) | 1.77 (1.04 - 3.01) | 1.83 (1.08 - 3.12) |
| 6339 | Supervisors and managers - catering and system catering | 187 | 11 | 10.41 (3.38) | 1.94 (1.04 - 3.62) | 1.96 (1.06 - 3.63) | 1.83 (0.99 - 3.39) |
| 6227 | Professions in the sale of motor vehicles, two-wheelers and accessories | 601 | 27 | 10.82 (2.75) | 1.81 (1.21 - 2.71) | 1.77 (1.18 - 2.66) | 1.77 (1.18 - 2.65) |
| 6131 | Professions in property marketing and management | 330 | 15 | 11.18 (2.24) | 1.78 (1.05 - 3.03) | 1.77 (1.04 - 3.01) | 1.73 (1.01 - 2.94) |
| 2739 | Supervisors and managers - Technical production planning and control | 746 | 47 | 11.40 (1.96) | 1.70 (1.24 - 2.32) | 1.70 (1.24 - 2.34) | 1.72 (1.25 - 2.36) |
| 5253 | Crane operators, lift operators and operators of related lifting equipment | 1066 | 75 | 10.66 (2.97) | 1.88 (1.45 - 2.44) | 1.85 (1.42 - 2.40) | 1.70 (1.31 - 2.21) |
| 5410 | Cleaning professions (without specialization) | 10060 | 678 | 10.69 (2.97) | 1.99 (1.76 - 2.25) | 1.98 (1.75 - 2.24) | 1.67 (1.47 - 1.89) |
| 5312 | Occupations in occupational safety and safety technology | 235 | 12 | 10.28 (3.39) | 1.79 (0.98 - 3.25) | 1.80 (0.99 - 3.27) | 1.67 (0.91 - 3.04) |
| 3430 | Occupations in supply and disposal (without specialization) | 842 | 62 | 11.09 (2.52) | 1.92 (1.45 - 2.54) | 1.86 (1.40 - 2.46) | 1.66 (1.25 - 2.20) |
| 9213 | Professions in customer management | 306 | 12 | 10.90 (2.69) | 1.64 (0.91 - 2.95) | 1.61 (0.89 - 2.91) | 1.61 (0.89 - 2.92) |
| 2210 | Professions in plastics and rubber production (without specialization) | 3083 | 180 | 10.96 (2.63) | 1.75 (1.46 - 2.10) | 1.76 (1.47 - 2.11) | 1.60 (1.34 - 1.92) |
| 8311 | Professions in childcare and education | 7151 | 278 | 11.39 (2.00) | 1.54 (1.32 - 1.79) | 1.54 (1.32 - 1.79) | 1.59 (1.37 - 1.86) |
| 2420 | Metalworking professions (without specialization) | 4622 | 264 | 10.73 (2.94) | 1.66 (1.41 - 1.94) | 1.66 (1.41 - 1.95) | 1.54 (1.31 - 1.81) |
| 2421 | Professions in non-cutting metalworking | 345 | 23 | 11.21 (2.30) | 1.64 (1.06 - 2.55) | 1.55 (1.00 - 2.42) | 1.47 (0.94 - 2.29) |
| 2442 | Professions in welding and joining technology | 962 | 52 | 11.03 (2.53) | 1.47 (1.09 - 1.98) | 1.49 (1.11 - 2.02) | 1.47 (1.09 - 1.98) |
| 8111 | Dental assistants | 1685 | 43 | 10.98 (2.63) | 1.52 (1.11 - 2.10) | 1.50 (1.09 - 2.06) | 1.46 (1.06 - 2.01) |
| 8312 | Professions in social work and social pedagogy | 1774 | 64 | 11.20 (2.32) | 1.30 (1.00 - 1.70) | 1.30 (0.99 - 1.70) | 1.45 (1.11 - 1.91) |
| 5131 | Professions in the warehousing industry | 16105 | 828 | 10.68 (2.97) | 1.59 (1.41 - 1.80) | 1.58 (1.40 - 1.78) | 1.44 (1.28 - 1.63) |
| 8231 | Hairdressing professions | 1465 | 44 | 10.53 (3.06) | 1.51 (1.10 - 2.08) | 1.52 (1.10 - 2.08) | 1.44 (1.05 - 1.98) |
| 7110 | Managing directors and board members | 424 | 22 | 11.17 (2.39) | 1.46 (0.94 - 2.27) | 1.50 (0.96 - 2.34) | 1.44 (0.92 - 2.25) |
| 8171 | Professions in physiotherapy | 931 | 32 | 11.29 (2.22) | 1.34 (0.93 - 1.94) | 1.38 (0.95 - 1.99) | 1.41 (0.97 - 2.03) |
| 5411 | Professions in building cleaning | 1506 | 83 | 10.52 (3.06) | 1.73 (1.36 - 2.21) | 1.67 (1.31 - 2.13) | 1.40 (1.10 - 1.79) |
| 2451 | Professions in precision engineering | 408 | 17 | 11.11 (2.64) | 1.43 (0.87 - 2.36) | 1.44 (0.87 - 2.38) | 1.40 (0.85 - 2.31) |
| 2822 | Professions in the manufacture of clothing, hats and caps | 465 | 23 | 11.19 (2.31) | 1.40 (0.91 - 2.16) | 1.46 (0.95 - 2.25) | 1.40 (0.91 - 2.16) |
| 2310 | Professions in paper and packaging technology (without specialization) | 304 | 15 | 10.51 (3.19) | 1.50 (0.88 - 2.55) | 1.54 (0.90 - 2.64) | 1.39 (0.81 - 2.38) |
| 2512 | Machine and plant operators | 2690 | 134 | 11.18 (2.37) | 1.46 (1.19 - 1.78) | 1.45 (1.18 - 1.77) | 1.38 (1.13 - 1.69) |
| 2422 | Professions in grinding metalworking | 445 | 25 | 11.25 (2.23) | 1.43 (0.94 - 2.18) | 1.42 (0.93 - 2.17) | 1.37 (0.90 - 2.10) |
| 3330 | Professions in finishing and drywall construction (without specialization) | 503 | 23 | 10.57 (3.03) | 1.50 (0.97 - 2.32) | 1.57 (1.01 - 2.42) | 1.37 (0.88 - 2.12) |
| 2630 | Professions in electrical engineering (without specialization) | 1976 | 91 | 10.87 (2.80) | 1.44 (1.14 - 1.82) | 1.46 (1.16 - 1.85) | 1.37 (1.08 - 1.73) |
| 5311 | Professions in property, asset and personal protection | 2442 | 144 | 10.69 (2.98) | 1.61 (1.32 - 1.96) | 1.50 (1.23 - 1.83) | 1.36 (1.12 - 1.66) |
| 8319 | Supervisors and managers - education, social work, curative education | 248 | 11 | 11.52 (1.72) | 1.26 (0.68 - 2.34) | 1.25 (0.67 - 2.33) | 1.36 (0.73 - 2.54) |
| 8321 | Professions in housekeeping | 2879 | 151 | 10.99 (2.62) | 1.52 (1.25 - 1.83) | 1.50 (1.24 - 1.82) | 1.36 (1.12 - 1.64) |
| 2930 | Cooks (without specialization) | 6208 | 285 | 10.40 (3.21) | 1.53 (1.32 - 1.78) | 1.55 (1.33 - 1.80) | 1.34 (1.15 - 1.57) |
| 2423 | Professions in metalworking | 2158 | 92 | 11.27 (2.21) | 1.29 (1.02 - 1.63) | 1.31 (1.04 - 1.66) | 1.34 (1.06 - 1.69) |
| 2529 | Supervisors and managers - vehicle, aerospace and shipbuilding technology | 230 | 11 | 11.02 (2.64) | 1.23 (0.66 - 2.29) | 1.27 (0.68 - 2.37) | 1.33 (0.72 - 2.48) |
| 2520 | Professions in automotive engineering (without specialization) | 259 | 12 | 10.71 (2.76) | 1.56 (0.86 - 2.83) | 1.50 (0.83 - 2.73) | 1.33 (0.73 - 2.41) |
| 8313 | Professions in curative education and special needs education | 1565 | 56 | 11.13 (2.35) | 1.28 (0.96 - 1.70) | 1.28 (0.96 - 1.70) | 1.32 (1.00 - 1.76) |
| 2220 | Professions in paint and varnish technology (without specialization) | 426 | 22 | 11.03 (2.51) | 1.41 (0.90 - 2.20) | 1.39 (0.89 - 2.17) | 1.32 (0.85 - 2.07) |
| 6241 | Professions in the sale of drugstore and pharmacy goods | 836 | 26 | 11.09 (2.56) | 1.33 (0.89 - 1.99) | 1.35 (0.90 - 2.02) | 1.32 (0.88 - 1.98) |
| 3110 | Professions in construction planning and supervision (without specialization) | 369 | 12 | 11.19 (2.28) | 1.14 (0.64 - 2.06) | 1.19 (0.66 - 2.14) | 1.32 (0.73 - 2.37) |
| 5162 | Forwarding and logistics clerks | 1098 | 40 | 10.82 (2.83) | 1.41 (1.01 - 1.97) | 1.36 (0.97 - 1.90) | 1.31 (0.94 - 1.84) |
| 4131 | Professions in chemical and pharmaceutical engineering | 1637 | 75 | 11.25 (2.29) | 1.35 (1.05 - 1.74) | 1.35 (1.05 - 1.74) | 1.30 (1.01 - 1.68) |
| 2234 | Professions in wood, furniture and interior design | 1436 | 59 | 11.14 (2.45) | 1.24 (0.94 - 1.65) | 1.28 (0.97 - 1.70) | 1.29 (0.98 - 1.72) |
| 8182 | Professions in pharmaceutical-technical assistance | 567 | 15 | 11.18 (2.53) | 1.23 (0.73 - 2.08) | 1.24 (0.73 - 2.10) | 1.29 (0.76 - 2.18) |
| 2341 | Professions in printing technology | 744 | 35 | 11.20 (2.28) | 1.30 (0.91 - 1.85) | 1.35 (0.94 - 1.92) | 1.29 (0.90 - 1.84) |
| 2510 | Professions in mechanical and operating engineering (without specialization) | 5184 | 227 | 11.13 (2.47) | 1.30 (1.10 - 1.54) | 1.31 (1.11 - 1.54) | 1.28 (1.08 - 1.51) |
| 6330 | Professions in catering service (without specialization) | 4744 | 172 | 9.97 (3.54) | 1.47 (1.23 - 1.76) | 1.46 (1.22 - 1.75) | 1.26 (1.05 - 1.51) |
| 2312 | Professions in paper processing and packaging technology | 415 | 22 | 11.53 (1.75) | 1.30 (0.84 - 2.03) | 1.30 (0.83 - 2.02) | 1.25 (0.80 - 1.96) |
| 5415 | Professions in vehicle cleaning | 360 | 16 | 10.34 (3.35) | 1.44 (0.86 - 2.41) | 1.45 (0.86 - 2.43) | 1.25 (0.74 - 2.10) |
| 3210 | Professions in building construction (without specialization) | 2683 | 119 | 10.61 (3.05) | 1.39 (1.12 - 1.71) | 1.42 (1.15 - 1.75) | 1.23 (1.00 - 1.52) |
| 7211 | Bank clerks | 1855 | 57 | 11.49 (1.96) | 1.16 (0.88 - 1.54) | 1.17 (0.88 - 1.55) | 1.23 (0.93 - 1.63) |
| 2824 | Professions in upholstery and vehicle interiors | 224 | 10 | 11.08 (2.37) | 1.29 (0.68 - 2.47) | 1.29 (0.67 - 2.48) | 1.22 (0.64 - 2.34) |
| 6112 | Professions in sales (except information and communication technologies) | 2265 | 76 | 10.98 (2.62) | 1.20 (0.93 - 1.54) | 1.20 (0.94 - 1.54) | 1.22 (0.95 - 1.57) |
| 2611 | Professions in mechatronics | 606 | 14 | 10.79 (2.93) | 1.28 (0.74 - 2.21) | 1.26 (0.73 - 2.17) | 1.22 (0.71 - 2.10) |
| 5212 | Professional drivers (goods transport/trucks) | 6884 | 384 | 11.01 (2.64) | 1.34 (1.16 - 1.55) | 1.31 (1.13 - 1.51) | 1.21 (1.05 - 1.40) |
| 7311 | Assistants in law firms and notary's offices | 606 | 14 | 10.85 (2.78) | 1.20 (0.70 - 2.07) | 1.20 (0.70 - 2.07) | 1.20 (0.70 - 2.07) |
| 2513 | Technical service staff in maintenance and repair | 1493 | 61 | 11.26 (2.31) | 1.21 (0.92 - 1.59) | 1.19 (0.90 - 1.57) | 1.19 (0.90 - 1.58) |
| 6231 | Professions in the sale of bakery and confectionery products | 1667 | 54 | 10.72 (2.90) | 1.29 (0.97 - 1.72) | 1.26 (0.94 - 1.69) | 1.19 (0.89 - 1.59) |
| 3420 | Professions in plumbing (without specialization) | 386 | 15 | 11.04 (2.65) | 1.18 (0.69 - 2.01) | 1.21 (0.71 - 2.07) | 1.17 (0.69 - 1.99) |
| 5133 | Professions in freight and goods handling | 240 | 12 | 11.48 (1.71) | 1.54 (0.85 - 2.79) | 1.31 (0.71 - 2.42) | 1.17 (0.63 - 2.16) |
| 2511 | Machine and equipment assemblers | 2024 | 83 | 11.05 (2.58) | 1.21 (0.95 - 1.54) | 1.23 (0.97 - 1.57) | 1.16 (0.91 - 1.48) |
| 5121 | Road and tunnel attendants | 520 | 25 | 11.57 (1.73) | 1.17 (0.77 - 1.77) | 1.11 (0.73 - 1.70) | 1.16 (0.76 - 1.77) |
| 2731 | Professions in technical quality assurance | 1372 | 54 | 11.17 (2.41) | 1.16 (0.87 - 1.55) | 1.16 (0.86 - 1.55) | 1.16 (0.86 - 1.55) |
| 4132 | Professions in the chemical-technical laboratory | 400 | 12 | 11.54 (1.81) | 1.15 (0.64 - 2.07) | 1.14 (0.63 - 2.06) | 1.16 (0.64 - 2.09) |
| 3119 | Supervisors and managers - construction planning and supervision, architecture | 264 | 11 | 11.16 (2.52) | 1.15 (0.62 - 2.14) | 1.13 (0.61 - 2.09) | 1.15 (0.62 - 2.13) |
| 5218 | Drivers in road transport (other specific job description) | 1588 | 72 | 10.12 (3.49) | 1.32 (1.02 - 1.71) | 1.27 (0.98 - 1.65) | 1.14 (0.88 - 1.48) |
| 7139 | Supervisory and management staff - Company organisation and strategy | 1416 | 47 | 11.16 (2.45) | 1.09 (0.80 - 1.49) | 1.12 (0.82 - 1.52) | 1.14 (0.84 - 1.55) |
| 3411 | Groundsmen and equipment attendants | 514 | 24 | 11.37 (2.07) | 1.13 (0.74 - 1.73) | 1.14 (0.74 - 1.74) | 1.13 (0.74 - 1.74) |
| 3212 | Jobs in the bricklaying trade | 1015 | 38 | 11.06 (2.56) | 1.11 (0.79 - 1.57) | 1.13 (0.80 - 1.59) | 1.13 (0.80 - 1.60) |
| 1171 | Professions in forestry | 339 | 13 | 10.93 (2.87) | 1.09 (0.62 - 1.93) | 1.14 (0.64 - 2.01) | 1.13 (0.64 - 2.00) |
| 2625 | Professions in electrical operating technology | 935 | 30 | 11.25 (2.37) | 1.17 (0.80 - 1.72) | 1.10 (0.74 - 1.62) | 1.12 (0.76 - 1.65) |
| 7322 | Administrative professions in the social and healthcare sector | 549 | 17 | 11.28 (2.23) | 1.09 (0.67 - 1.80) | 1.08 (0.66 - 1.78) | 1.11 (0.68 - 1.83) |
| 1110 | Professions in agriculture (without specialization) | 1127 | 34 | 10.81 (2.76) | 1.21 (0.84 - 1.73) | 1.22 (0.85 - 1.75) | 1.10 (0.77 - 1.58) |
| 5252 | Operators of earthmoving and related machinery | 679 | 34 | 11.13 (2.53) | 1.22 (0.85 - 1.75) | 1.15 (0.80 - 1.65) | 1.10 (0.76 - 1.58) |
| 2412 | Professions in metal forming | 459 | 21 | 11.13 (2.43) | 1.15 (0.73 - 1.81) | 1.13 (0.72 - 1.79) | 1.09 (0.69 - 1.72) |
| 6230 | Professions in the sale of food (without specialization) | 1063 | 29 | 10.56 (3.07) | 1.15 (0.78 - 1.69) | 1.17 (0.80 - 1.72) | 1.07 (0.73 - 1.57) |
| 2621 | Professions in building electrics | 1896 | 62 | 11.11 (2.53) | 1.05 (0.80 - 1.39) | 1.05 (0.80 - 1.39) | 1.06 (0.81 - 1.40) |
| 3222 | Professions in road and asphalt construction | 455 | 17 | 11.35 (2.14) | 1.12 (0.68 - 1.85) | 1.07 (0.65 - 1.77) | 1.04 (0.63 - 1.72) |
| 6210 | Professions in sales (without product specialization) | 9974 | 278 | 10.84 (2.78) | 1.10 (0.94 - 1.28) | 1.10 (0.94 - 1.28) | 1.03 (0.88 - 1.19) |
| 6322 | Professions in hotel service | 1561 | 42 | 10.29 (3.29) | 1.14 (0.83 - 1.58) | 1.16 (0.84 - 1.60) | 1.02 (0.74 - 1.42) |
| 6219 | Supervisory and management staff - Sales | 579 | 16 | 11.12 (2.59) | 1.02 (0.61 - 1.69) | 1.04 (0.62 - 1.72) | 1.02 (0.61 - 1.70) |
| 9133 | Professions in educational science | 369 | 10 | 10.95 (2.56) | 0.96 (0.50 - 1.82) | 0.97 (0.51 - 1.83) | 1.02 (0.53 - 1.93) |
| 3410 | Professions in building services engineering (without specialization) | 2721 | 132 | 11.14 (2.44) | 1.09 (0.89 - 1.33) | 1.07 (0.87 - 1.31) | 1.01 (0.83 - 1.24) |
| 7221 | Professions in accounting | 1590 | 47 | 11.33 (2.13) | 0.97 (0.71 - 1.31) | 0.97 (0.71 - 1.32) | 1.01 (0.74 - 1.37) |
| 2452 | Professions in tool technology | 865 | 26 | 11.23 (2.37) | 0.94 (0.63 - 1.41) | 0.97 (0.65 - 1.46) | 1.01 (0.67 - 1.51) |
| 2631 | Professions in information and telecommunications technology | 890 | 25 | 11.05 (2.51) | 0.99 (0.66 - 1.50) | 0.99 (0.66 - 1.50) | 1.01 (0.67 - 1.52) |
| 2722 | Professions in construction and appliance manufacturing | 470 | 12 | 11.40 (2.13) | 0.90 (0.50 - 1.61) | 0.92 (0.51 - 1.65) | 1.01 (0.56 - 1.81) |
| 6211 | Cashiers and ticket sellers | 1069 | 33 | 10.79 (2.86) | 1.06 (0.74 - 1.52) | 1.04 (0.73 - 1.50) | 0.99 (0.69 - 1.42) |
| 2430 | Professions in metal surface treatment (without specialization) | 601 | 24 | 11.05 (2.53) | 1.11 (0.73 - 1.70) | 1.09 (0.72 - 1.67) | 0.99 (0.65 - 1.51) |
| 3220 | Professions in civil engineering (without specialization) | 710 | 24 | 11.05 (2.58) | 1.03 (0.68 - 1.58) | 1.06 (0.70 - 1.63) | 0.98 (0.64 - 1.50) |
| 9999 | Other occupations | 30486 | 893 | 11.01 (2.61) | 0.99 (0.88 - 1.11) | 0.99 (0.88 - 1.11) | 0.98 (0.87 - 1.11) |
| 2230 | Occupations in woodworking and wood processing (without specialization) | 683 | 27 | 10.96 (2.65) | 1.04 (0.70 - 1.55) | 1.05 (0.70 - 1.58) | 0.98 (0.65 - 1.47) |
| 5132 | Professions for postal and delivery services | 2159 | 79 | 10.56 (3.16) | 1.16 (0.91 - 1.48) | 1.14 (0.89 - 1.45) | 0.96 (0.75 - 1.23) |
| 3214 | Professions in the roofing industry | 433 | 11 | 11.04 (2.59) | 0.94 (0.51 - 1.73) | 0.98 (0.53 - 1.81) | 0.96 (0.52 - 1.77) |
| 2730 | Professions in technical production planning and control | 1234 | 43 | 11.26 (2.33) | 0.99 (0.72 - 1.36) | 0.96 (0.69 - 1.32) | 0.95 (0.69 - 1.32) |
| 6221 | Professions in the sale of clothing, sporting goods, leather goods and shoes | 1244 | 31 | 10.71 (2.91) | 0.98 (0.68 - 1.42) | 1.01 (0.70 - 1.46) | 0.95 (0.66 - 1.38) |
| 3431 | Professions in water supply and wastewater technology | 320 | 11 | 11.37 (2.19) | 0.90 (0.49 - 1.67) | 0.93 (0.50 - 1.71) | 0.94 (0.51 - 1.74) |
| 2441 | Professions in metal construction | 2810 | 92 | 11.05 (2.57) | 0.95 (0.76 - 1.20) | 0.96 (0.76 - 1.21) | 0.94 (0.74 - 1.19) |
| 2522 | Professions in agricultural and construction machinery technology | 460 | 12 | 11.28 (2.35) | 0.94 (0.52 - 1.69) | 0.94 (0.52 - 1.69) | 0.94 (0.52 - 1.69) |
| 7230 | Professions in tax consultancy | 1148 | 23 | 11.38 (2.00) | 0.88 (0.58 - 1.35) | 0.89 (0.58 - 1.36) | 0.93 (0.61 - 1.42) |
| 7130 | Professions in commercial and technical business administration (without specialization) | 5690 | 133 | 11.11 (2.50) | 0.91 (0.75 - 1.11) | 0.91 (0.75 - 1.11) | 0.92 (0.76 - 1.13) |
| 7152 | Professions in personnel services | 529 | 11 | 10.93 (2.77) | 0.87 (0.47 - 1.60) | 0.85 (0.46 - 1.57) | 0.92 (0.50 - 1.69) |
| 4310 | Professions in computer science (without specialization) | 743 | 16 | 11.10 (2.52) | 0.93 (0.56 - 1.55) | 0.90 (0.54 - 1.51) | 0.90 (0.54 - 1.51) |
| 2521 | Professions in automotive engineering | 3124 | 77 | 11.11 (2.56) | 0.90 (0.70 - 1.15) | 0.90 (0.70 - 1.16) | 0.90 (0.70 - 1.15) |
| 3421 | Professions in sanitary, heating and air-conditioning technology | 1681 | 45 | 11.16 (2.51) | 0.88 (0.65 - 1.21) | 0.89 (0.65 - 1.22) | 0.88 (0.64 - 1.21) |
| 1210 | Professions in horticulture (without specialization) | 1449 | 50 | 10.88 (2.80) | 0.96 (0.71 - 1.29) | 0.94 (0.70 - 1.28) | 0.84 (0.62 - 1.14) |
| 7321 | Professions in social administration and insurance | 1492 | 33 | 11.68 (1.51) | 0.79 (0.55 - 1.13) | 0.79 (0.55 - 1.14) | 0.83 (0.58 - 1.20) |
| 6232 | Professions in the sale of meat products | 754 | 19 | 10.88 (2.79) | 0.85 (0.54 - 1.36) | 0.85 (0.53 - 1.36) | 0.83 (0.52 - 1.33) |
| 6121 | Wholesale and foreign trade clerks | 992 | 17 | 11.08 (2.49) | 0.85 (0.52 - 1.39) | 0.84 (0.51 - 1.38) | 0.83 (0.51 - 1.36) |
| 6111 | Professions in purchasing | 608 | 13 | 11.31 (2.25) | 0.76 (0.44 - 1.34) | 0.79 (0.45 - 1.38) | 0.83 (0.47 - 1.45) |
| 2922 | Professions in bakery and confectionery production | 779 | 18 | 11.04 (2.55) | 0.87 (0.54 - 1.40) | 0.86 (0.53 - 1.39) | 0.81 (0.50 - 1.31) |
| 2321 | Professions in digital and print media design | 513 | 12 | 11.17 (2.35) | 0.85 (0.47 - 1.52) | 0.84 (0.47 - 1.52) | 0.81 (0.45 - 1.45) |
| 3321 | Professions for painting and varnishing work | 1255 | 29 | 10.65 (3.09) | 0.77 (0.52 - 1.13) | 0.80 (0.55 - 1.18) | 0.77 (0.53 - 1.13) |
| 9212 | Professions in dialogue marketing | 906 | 16 | 10.60 (3.10) | 0.82 (0.50 - 1.37) | 0.80 (0.48 - 1.33) | 0.76 (0.46 - 1.27) |
| 4341 | Professions in software development | 765 | 11 | 11.24 (2.22) | 0.64 (0.35 - 1.17) | 0.65 (0.35 - 1.18) | 0.68 (0.37 - 1.25) |
| 2721 | Technical draughtsmen/draftswomen | 731 | 11 | 11.24 (2.26) | 0.61 (0.33 - 1.11) | 0.61 (0.33 - 1.11) | 0.63 (0.34 - 1.15) |
| 1214 | Professions in gardening, landscaping and sports ground construction | 649 | 13 | 11.06 (2.60) | 0.62 (0.36 - 1.09) | 0.63 (0.36 - 1.11) | 0.59 (0.34 - 1.04) |
| 6226 | Professions in the garden, DIY, pet and pet supplies sales | 596 | 10 | 11.22 (2.35) | 0.57 (0.30 - 1.08) | 0.58 (0.31 - 1.09) | 0.58 (0.31 - 1.10) |

1: adjusted for sex and age

2: adjusted for sex, age, and number of comorbidities

3: adjusted for sex, age, number of comorbidities and SES (vocational education and GISD)

Supplementary Table S6. Sensitivity analysis: risk of hospitalization due to COVID-19 by occupation, with further adjustment by status of vocational training among two different age groups (no vocational training [<30 years], no vocational training [≥30 years])

| **Occupational category** | **KldB 2010** | **N (total)** | **N (cases)** | **Person-months (SD)** | **HR* (95% CI)** |
| --- | --- | --- | --- | --- | --- |
| Business administration and related services | 714x, 732x, !7321, !7322 | 15628 | 457 | 11.19 (2.39) | 1 (Reference) |
| Occupations in theology and church work | 8331, 8332, 8338 | 209 | 23 | 11.15 (2.19) | 3.04 (1.92 - 4.82) |
| Occupations in healthcare | 5332, 5334, 8110, 8111, 8113, 8118, 8122, 8123, 8130, 8131,  8132, 8133, 8134, 8135, 8138, 8140, 8141, 8142, 8143, 8144, 8145, 8146, 8147, 8148, 8149, 8163, 8171, 8172, 8173, 8174,  8175, 8176, 8177, 8178, 8179,  8180, 8182, 8188, 8210, 8218, 8223 | 24152 | 1606 | 11.09 (2.49) | 2.74 (2.46 - 3.05) |
| Bus and tram drivers | 5213 | 1663 | 182 | 11.01 (2.57) | 2.46 (2.03 - 2.97) |
| Occupations in meat processing | 2923 | 704 | 46 | 10.76 (2.78) | 2.15 (1.56 - 2.97) |
| Professional drivers in passenger transport | 5211 | 1172 | 109 | 9.93 (3.56) | 2.00 (1.59 - 2.51) |
| Occupations in property marketing and management | 6131 | 330 | 15 | 11.18 (2.24) | 1.74 (1.02 - 2.96) |
| Occupations in social sector | 8311, 831 , 8313, 831 , 8315 | 11302 | 461 | 11.28 (2.16) | 1.61 (1.41 - 1.84) |
| Occupations in the laboratory | 8121, 8254 | 603 | 24 | 11.35 (2.09) | 1.57 (1.03 - 2.40) |
| Service staff in passenger transport | 5140, 5141, 5142, 5143 | 333 | 16 | 11.08 (2.49) | 1.50 (0.89 - 2.52) |
| Occupations in personal care | 8231, 8232, 8233, 8234 | 1742 | 51 | 10.51 (3.08) | 1.39 (1.03 - 1.87) |
| Occupations in housekeeping and domestic help | 8321 | 2879 | 151 | 10.99 (2.62) | 1.35 (1.11 - 1.63) |
| Occupations in gastronomy | 6330, 6331, 6332 | 5189 | 196 | 9.98 (3.53) | 1.31 (1.10 - 1.56) |
| Bank clerks | 7211 | 1855 | 57 | 11.49 (1.96) | 1.23 (0.93 - 1.63) |
| Teaching and training occupations | 84xx | 2542 | 76 | 10.79 (2.85) | 1.19 (0.92 - 1.53) |
| Other occupations |  | 167740 | 7067 | 10.95 (2.68) | 1.13 (1.02 - 1.25) |
| Occupations in sales | 62xx, !6219 | 19139 | 544 | 10.83 (2.80) | 1.04 (0.92 - 1.18) |
| Tourist guides and occupations in hotel service | 6314 6322 | 1568 | 42 | 10.30 (3.28) | 1.02 (0.74 - 1.41) |
| Occupations in postal and delivery services | 5132 | 2159 | 79 | 10.56 (3.16) | 0.96 (0.75 - 1.23) |

*adjusted for age, sex, number of comorbidities, German Index of Socioeconomic Deprivation, educational status (no vocational training [<30 years], no vocational training [≥30 years], vocational training, degree program, unknown status)

Supplementary Table S7. Sensitivity analysis: risk of hospitalization due to COVID-19 by occupation, with replacement of missing educational qualification in year 2020 by information from years 2011-2019

| **Occupational category** | **KldB 2010** | **N (total)** | **N (cases)** | **Person-months (SD)** | **HR* (95% CI)** |
| --- | --- | --- | --- | --- | --- |
| Business administration and related services | 714x, 732x, !7321, !7322 | 15628 | 457 | 11.19 (2.39) | 1 (Reference) |
| Occupations in theology and church work | 8331, 8332, 8338 | 209 | 23 | 11.15 (2.19) | 3.04 (1.92 - 4.80) |
| Occupations in healthcare | 5332, 5334, 8110, 8111, 8113, 8118, 8122, 8123, 8130, 8131,  8132, 8133, 8134, 8135, 8138, 8140, 8141, 8142, 8143, 8144, 8145, 8146, 8147, 8148, 8149, 8163, 8171, 8172, 8173, 8174,  8175, 8176, 8177, 8178, 8179,  8180, 8182, 8188, 8210, 8218, 8223 | 24152 | 1606 | 11.09 (2.49) | 2.76 (2.48 - 3.08) |
| Bus and tram drivers | 5213 | 1663 | 182 | 11.01 (2.57) | 2.51 (2.08 - 3.03) |
| Occupations in meat processing | 2923 | 704 | 46 | 10.76 (2.78) | 2.16 (1.57 - 2.98) |
| Professional drivers in passenger transport | 5211 | 1172 | 109 | 9.93 (3.56) | 2.04 (1.63 - 2.57) |
| Occupations in property marketing and management | 6131 | 330 | 15 | 11.18 (2.24) | 1.73 (1.01 - 2.94) |
| Occupations in social sector | 8311, 831 , 8313, 831 , 8315 | 11302 | 461 | 11.28 (2.16) | 1.61 (1.41 - 1.83) |
| Occupations in laboratory | 8121, 8254 | 603 | 24 | 11.35 (2.09) | 1.57 (1.03 - 2.40) |
| Service staff in passenger transport | 5140, 5141, 5142, 5143 | 333 | 16 | 11.08 (2.49) | 1.55 (0.92 - 2.60) |
| Occupations in personal care | 8231, 8232, 8233, 8234 | 1742 | 51 | 10.51 (3.08) | 1.41 (1.05 - 1.90) |
| Occupations in housekeeping and domestic help | 8321 | 2879 | 151 | 10.99 (2.62) | 1.36 (1.12 - 1.65) |
| Occupations in gastronomy | 6330, 6331, 6332 | 5189 | 196 | 9.98 (3.53) | 1.33 (1.12 - 1.58) |
| Bank clerks | 7211 | 1855 | 57 | 11.49 (1.96) | 1.21 (0.92 - 1.61) |
| Other occupations |  | 167740 | 7067 | 10.95 (2.68) | 1.14 (1.03 - 1.26) |
| Teaching and training occupations | 84xx | 2542 | 76 | 10.79 (2.85) | 1.12 (0.87 - 1.44) |
| Occupations in sales | 62xx, !6219 | 19139 | 544 | 10.83 (2.80) | 1.05 (0.92 - 1.19) |
| Tourist guides and occupations in hotel service | 6314 6322 | 1568 | 42 | 10.30 (3.28) | 1.03 (0.74 - 1.42) |
| Occupations in postal and delivery services | 5132 | 2159 | 79 | 10.56 (3.16) | 0.97 (0.75 - 1.24) |

*adjusted for age, sex, number of comorbidities, German Index of Socioeconomic Deprivation, educational status (missing 2020 information replaced by information from the years 2011-2019

Supplementary Figure S1


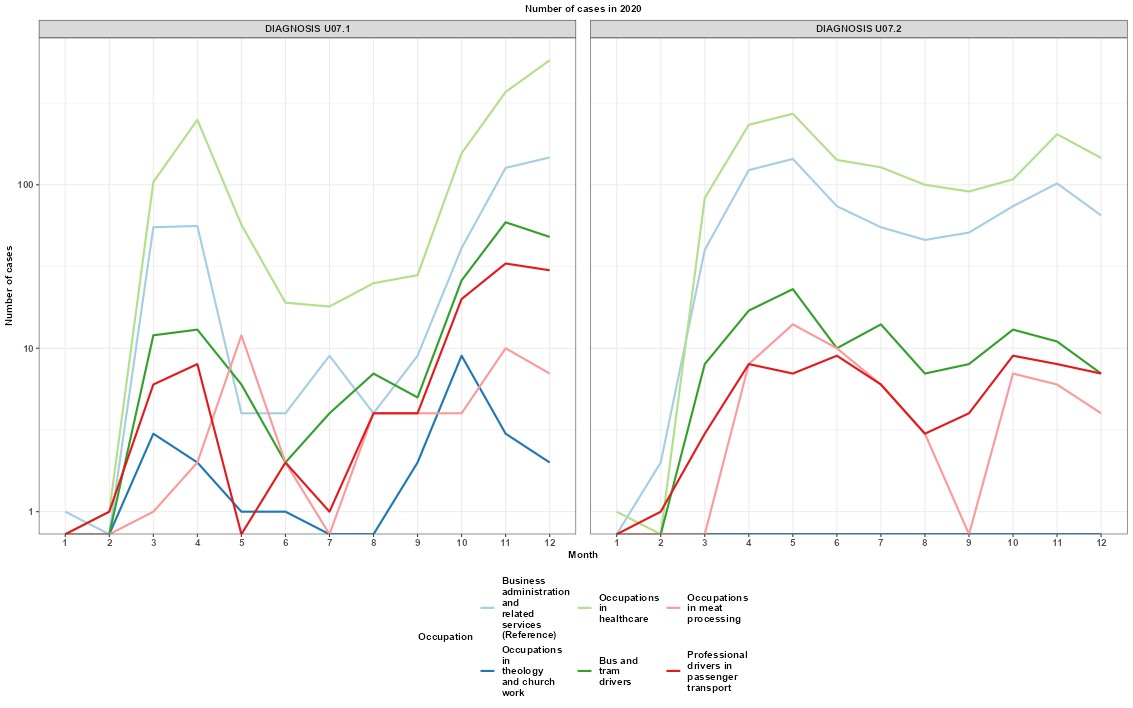

Supplement: Supplementary file 1 — Supplementary Material 1 [file 12889_2024_20706_MOESM1_ESM.docx]
